# IL28B *rs12979860* polymorphism and zinc supplementation affect treatment outcome and liver fibrosis after direct-acting antiviral hepatitis C therapy

**DOI:** 10.1186/s43141-021-00250-y

**Published:** 2021-10-08

**Authors:** Abdelfattah M. Attallah, Dalia Omran, Mohamed A. Abdelrazek, Mohamed Hassany, Sameh Saif, Aza Farid, Riham El Essawey, Muhammad Abdel Ghaffar, Marwa Aabdelghany, Ayman Yosry

**Affiliations:** 1 Biotechnology Research Center, P.O. Box (14), 23 July St., Industrial Zone, New Damietta City, 34517 Egypt; 2 Faculty of Medicine, Cairo University, Cairo, Egypt; 3 National Hepatology and Tropical Medicine Research Institute, Cairo, Egypt; 4 Hepato-Gastroenterology and Infectious Diseases Department, Ahmed Maher Teaching Hospital, Cairo, Egypt

**Keywords:** Direct-acting antiviral agents, Hepatitis C virus, Interleukin 28B, Liver fibrosis, Sustained virologic response

## Abstract

**Background:**

Impact of interleukin 28B (IL28B) *rs12979860* polymorphism on response to direct-acting antivirals agents in HCV genotype 4-infected patients is under investigation. Zinc may have an advantage in improvement of liver damage and treatment outcome. We aimed to evaluate IL28B polymorphism and zinc administration impact on patient response to treatment and amelioration of liver fibrosis.

**Results:**

Three hundred patients on anti-HCV treatments were equally categorized into patients treated with dual therapy (sofosbuvir/ribavirin) for 24 weeks, triple therapy (sofosbuvir/ribavirin+pegylated interferon-alpha) for 12 weeks, dual therapy plus oral zinc and with triple therapy plus oral zinc. All patients were genotyped for IL28B. Sustained virologic response (SVR) was achieved in 100% of patients with CC genotypes while 15.5% of CT/TT carriers did not attain SVR. After treatment, patients with CC genotype showed improvement in liver-related parameters compared with CT/TT genotypes. Zinc supplementation was associated with improved SVR in CT/TT genotypes and liver parameters in both CC and CT/TT genotypes. Hepatic fibrosis was improved in higher percent of CC genotype (16.7%) compared with CT/TT genotypes (5.8%). Interestingly with zinc administration, improved fibrosis increased to 60.9% in CC genotype *vs.* 15.4% in CT/TT genotypes.

**Conclusion:**

Absolute SVR rates in patients with IL28B CC genotype support their selection for shorter treatment duration and therefore associated with high economic value. IL28B polymorphism is associated with improvement of hepatic functions and fibrosis after antiviral treatments. Zinc is powerful supplement not only to increase SVR in non-responders but also to improve hepatic functions and fibrosis.

## Background

HCV infection eradication seems feasible owing to the significant advance made in the development and incorporation of new curative methods, especially direct-acting antivirals agents (DAAs) targeting different enzymes of the virus [1]. Addition of DAAs to pegylated interferon-alpha (Peg-IFN) and ribavirin (RBV) has resulted in improved sustained virologic response (SVR) rates. There are treatment challenges with these IFN-based regimens owing to adverse reactions and drug interactions [2]. Moreover, lower SVR rates with DAAs/INF combinations remain associated with some viral and host factors including high HCV viral loads, black race, IL28B CT/TT genotypes, advanced liver fibrosis, and prior HCV treatment experience [2, 3].

In treatment regimens including sofosbuvir (SOF), the response rates of patients with non-CC IL28B genotypes come closer to the response rates of patients with CC genotype.^4^ However, the IL28B variant still influences treatment outcomes [4, 5]. Studies are still now stratified according to IL28B genotype in the era of DAAs in attempt to improve treatment by shortening the duration in favorable IL28B genotypes [6]. Some of patients do not respond to therapy and are still viremic and returning to their societies as viral transmitters [7]. So, exploring patient factors contributing to relapse at the end of treatment are needed. It is unfortunate that there are limited data regarding the association between IL28B *rs12979860* single-nucleotide polymorphism (SNP) and response to treatment regimens including SOF in Egyptian patients with HCV genotype 4 [8].

Zinc deficiency increases susceptibility to delayed recovery from many pathogens as it plays a significant role in immune function [9]. Zinc signaling and homeostasis are required for immune response. Any alteration in zinc homeostasis is associated with chronic disease development [10]. Zinc deficiency due to interferon was reported for the first time by Gainer [11]. Then, other authors have reported that administration of zinc plays serious role in CHC outcome [9]. Thus, zinc administration with the standard HCV regimen may have an advantage in CHC patients’ pharmacotherapy [9]. Investigations concerning the effect of zinc supplementation on response to antiviral treatments and on liver damage improvement after treatment are needed.

The present study aimed to evaluate the association between IL28B *rs12979860* SNP and Egyptian patients’ response to treatment with SOF/RBV or SOF/RBV + Peg-INF in addition to amelioration of liver damage and fibrosis at the end of treatment. Also, this study aimed to assess the effect of oral administration of zinc on improvement of both SVR and amelioration of liver damage and fibrosis.

## Methods

### Patients

The study included 300 subjects randomly selected from candidates of direct-acting antiviral drugs at the National Hepatology and Tropical Medicine Research Institute, Cairo, Egypt, during 2015–2016. Patients were treated with either SOF + RBV or SOF + RBV + Peg-IFN regimens according to regulations put by the National Committee for Control of Viral Hepatitis (NNCVH) that was established by Ministry of Health to face Hepatitis C in Egypt.

The study design met the following inclusion and exclusion criteria. The criteria for inclusion were as follows: all eligible cases for treatment according to international guidelines (Child A, Child B), HCV-RNA-positive PCR, both sexes, ages above 18 years, and complete blood picture within normal ranges. Those found ineligible for the treatment were excluded from the study [age under 18, active substance abuse, untreated thyroid disease, history of malignancy, and hepatic decompensation [(Child C, (albumin< 2.8 g/dl, total serum bilirubin > 3 mg/dl, INR > 1.3), platelets < 50,000/mm^3^], presence of ascites on ultrasound, current or planned pregnancy, retinopathy, uncontrolled diabetes (HbA1C > 9%), serum creatinine > 1.5 mg/mL or end-stage renal disease, alcohol abuse, WBC < 3000/cmm, hemoglobin < 10 g/dl, hepatocellular carcinoma (HCC), and obese patients (body mass index > 35)]. Those patients were eligible for IFN. So, they received SOF + RBV for 12 weeks. Difficult to treat patients included are those having albumin ≤ 3.5 g/dL, bilirubin > 1.2 mg/dL, FIB-4 > 3.25, INR > 1.2, and platelet count < 150,000/mm^3^. This group was treated with SOF + RBV for 24 weeks. SOF was given at 400 mg oral daily dose. RBV was given in 2 divided daily oral doses adjusted to body weight (800, 1000, 1200, and 1400 mg for weights of < 50, 50–65, 65–80, and > 80 kg, respectively). Peg-IFN-α-2a was given in weekly doses (180 μg/week). The majority of patients had zinc deficiency with mean baseline level 55.4 ± 28.6 μg/dl. The clear outcome was defined as SVR (undetectable HCV-RNA 24 weeks after the treatment).

Patients were equally categorized into four groups (75 patients/group): group 1 included 75 CHC patients who received dual therapy comprising of SOF and RBV for duration of 24 weeks. Group 2 included 75 patients who received triple therapy comprising of Peg-IFN-α with SOF and RBV for duration of 12 weeks. In addition, to assess the effect of zinc supplementation on viral response, groups 3 and 4 were designed to include 75 patients received dual therapy or triple therapy; respectively with administration of zinc (two oral doses of zinc sulfate 440 mg/day). One of the patients in group 1, three in group 2, and three in group 3 were lost to follow-up neither by home visits nor telephone. Missing data of these patients were excluded from further analysis. Response to therapy was evaluated every month during the trial and 24 weeks after the end of treatment. A written informed consent was obtained from all participants before enrolment in the study and this work protocol was approved in accordance with the declaration of Helsinki Ethics.

### Clinical and laboratory examination

All patients were tested positive for HCV seromarkers: anti-HCV antibodies (Biomedica, Sorin, Italy) and HCV-RNA (COBAS Ampliprep/ COBAS TaqMan, Roche Diagnostics, Pleasanton, USA). All patients were subjected to laboratory tests including complete blood count using a hematology analyzer (Sysmex Corporation, Kobe, Japan) and liver function tests using an automated biochemistry analyzer (A15, Biosystem, Spain). All patients underwent measurement of liver stiffness using Fibroscan.

### Liver stiffness measurement

FibroScan™ (Echosens, Paris, France) was performed by trained clinicians according to previously described technique [12]. At each assessment, a total of ten measurements were obtained and the median, in kPa, was determined. Fibrosis stages were estimated from stiffness values: F0-F1: 2.5–6.9 kPa; F2: 7.0–9.4 kPa; F3: 9.5–12·4 kPa; F4: ≥ 12.5 kPa. Liver stiffness was assessed for two time intervals: pre-treatment and ≥ 24 weeks after the end of treatment.

### IL-28B *rs12979860* polymorphism identification

From whole blood samples of all subjects, genomic DNA was extracted using commercially DNA extraction kit (Roche Diagnostics GmbH, Mannheim, Germany). As previously described [13], IL-28B *rs12979860* genotyping was performed by a restriction fragment length polymorphism assay. Amplified product was obtained using the forward primer 5′-GCGGAAGGAGCAGTTGCGCT-3′ and the reverse primer 5′-GGGCTTTGCTGGGGGAGTG-3′ in a total volume of 20 μL containing 10 pmol/reaction of each primer, 125 μmol each of deoxynucleotide triphosphates, and Taq DNA polymerase 2 U/reaction (Fermentas, Thermo Fisher Scientific, Boston, MA, USA). The PCR cycles were as follows: 35 cycles of denaturation (at 95 °C for 30 s), annealing (at 62 °C for 30 s), and elongation (at 72 °C for 30 s) using Veriti 96-well thermal cycler (Applied Biosystems, Carlsbad, CA, USA). In a total volume of 20 μL, 10 ml of amplified product was digested with 5 U of the BstU-I restriction endonuclease (New England Biolabs, Ipswich, MA, USA) at 60 °C overnight. The digested fragments were 196 bp and 45 bp for CC genotype; 241 bp, 196 bp, and 45 bp for CT; and 241 bp for TT genotype. The fragments were electrophoresed in an ethidium bromide-stained 3.0% agarose gel and visualized by a gel doc unit (Bio-Rad, Hercules, CA, USA).

### Statistical analysis

Quantitative data were expressed and reported as mean ± standard deviation (SD) and compared using unpaired Student’s *t* test, ANOVA, and Mann-Whitney test as appropriate. Qualitative variables were expressed by numbers or percent and compared using chi-square (*χ*^2^) test or Fisher exact test. All tests were two-tailed and *P* value was considered to be significant if < 0.05. Patients’ data were analyzed using GraphPad Prism package v.5.0 (GraphPad Software, San Diego, CA) and SPSS software v.17.0 (SPSS Inc., Chicago, IL).

## Results

### Demographic characteristics between patients with different IL28B alleles

Of 293 patients (7 lost to follow-up) genotyped for *rs12979860* IL28 SNP, 93 were with CC genotype and 200 were T allele carriers (with CT/TT genotypes). There were no significant differences (*P* > 0.05) between patients with CC genotype and T allele carriers in the mean ages (SD) (52.9 (9.3) *vs.* 53.4 (8.5) years, respectively) and gender distribution (male/female) (55/39 *vs.* 120/80, respectively).

### Effect of IL28B C/T SNP and zinc administration on response to antiviral therapies

**Fig. 1:**
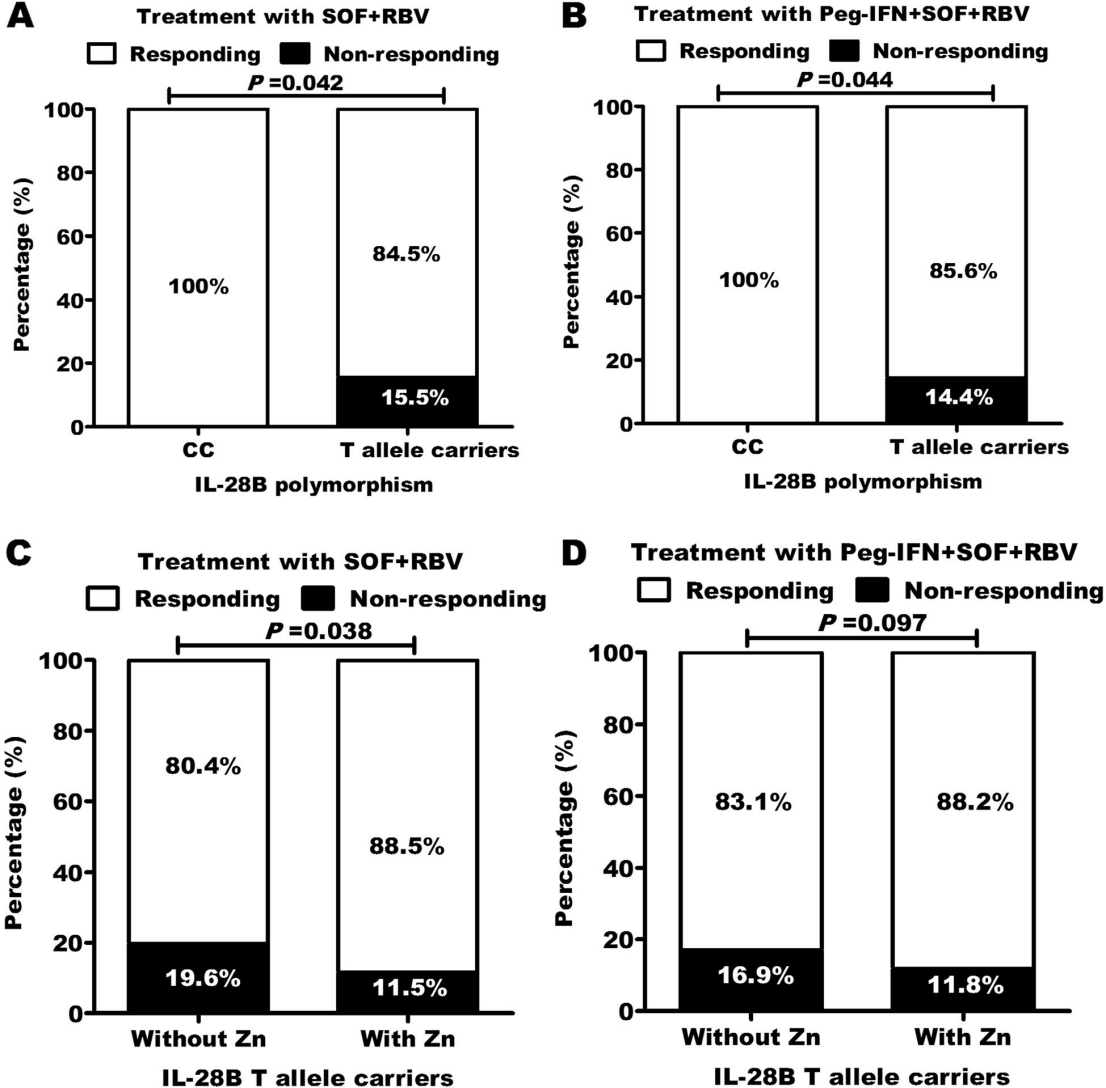
IL28B rs12979860 single-nucleotide polymorphism is linked to virologic response after treatment with direct-acting antiviral plus pegylated interferon/ribavirin. All patients (100%) with CC genotype achieved HCV-RNA < 25 IU/ml response after treatment with SOF/RBV with and without Peg-INF. In return, T allele carriers achieved HCV-RNA < 25 IU/ml response in **A** 84.5% after treatment with SOF/RBV and **B** 85.6% after treatment with SOF/RBV + Peg-INF. Administration of zinc improved virologic response after treatment with both **C** SOF/RBV and **D** SOF/RBV + Peg-INF. IL-28B *rs12979860* single-nucleotide polymorphism genotypes. T allele carriers = CT/TT combined

Overall, SVR was achieved in 100% of patients with CC genotypes in both SOF/RBV and SOF/RBV + Peg-INF therapeutic regimens. In T allele carriers (CT/TT), 15.5% and 14.4% did not attain SVR in SOF/RBV and SOF/RBV + Peg-INF regimens, respectively. The observed difference in SVR was significant (*P* = 0·04; Fig. 1A, B). In T allele carriers, zinc supplementation improved SVR to the therapeutic regimens. The rate of SVR was significantly higher in patients received zinc as compared to patients without zinc administration (Fig. 1C, D).

### CC genotype and zinc administration were linked with improved biochemical parameters

In both dual and triple therapeutic regimens, basal levels of liver enzyme activity, bilirubin, and albumin did not significantly improve in patients with IL-28B CT/TT genotypes. Patients with CC genotype showed a significant improvement in AST activity (*P* = 0·01) and albumin levels (*P* = 0.027) in patients treated with SOF/RBV and in AST activity (*P* = 0·032) and bilirubin levels (*P* = 0·033) in patients treated with SOF/RBV + Peg-INF (Table 1). Over all, oral zinc supplementation increased the improvement in liver parameters in both CC genotype and T allele carriers (Table 1). In patients with CC genotype, oral zinc caused significant (*P* < 0·05) improvement in ALT, AST, bilirubin, and albumin in patients treated with SOF/RBV or SOF/RBV + Peg-INF. Concerning CT/TT genotype, zinc supplementation caused significant improvement in bilirubin (*P* = 0·013) and albumin (*P* = 0·041) in patients treated with SOF/RBV. ALT (*P* = 0·024), AST (*P* = 0·009), and albumin (*P* = 0·004) were improved with zinc supplement in patients treated with SOF/RBV + Peg-INF.

**Table 1:** Patient’s characteristics before and after antiviral treatments

**Treatment with sofosbuvir and ribavirin**						
**Variables**	^*^IL-28B CC genotype		***P*** **value**	^*^IL-28B T allele carriers		***P*** **value**
	**Before**	**After**		**Before**	**After**	
**Without zinc administration**						
ALT (U/L)	48·4 ± 10·3	42·50 ± 11·6	0·184	62·5 ± 15·4	49·1 ± 13·3	0·164
AST (U/L)	46·6 ± 10·1	37·3 ± 9·1	0·010	75·2 ± 16·4	57·2 ± 13·3	0·088
Bilirubin (mg/dL)	1·0 ± 0·2	0·9 ± 0·15	0·885	1·1 ± 0·2	0·93 ± 0·12	0·363
Albumin (g/dL)	3·6 ± 0·98	4·3 ± 1·1	0·027	3·4 ± 0·88	3·7 ± 1·1	0·349
WBCs (× 10^9^/L)	4·6 ± 1·2	4·7 ± 0·7	0·961	4·7 ± 0·9	5·1 ± 1·3	0·465
Platelet count (×10^9^/L)	185·2 ± 30·1	157·5 ± 29·9	0·461	157·8 ± 20·3	160·0 ± 23·1	0·946
**With zinc administration**						
ALT (U/L)	41·8 ± 10·9	36·8 ± 9·8	0·060	38·0 ± 10·2	34·3 ± 9·7	0·382
AST (U/L)	42·5 ± 9·3	35·4 ± 9·2	0·004	38·3 ± 10·1	35·3 ± 10·2	0·361
Bilirubin (mg/dL)	1·1 ± 0·21	0·6 ± 0·15	0·0001	1·2 ± 0·2	0·8 ± 0·1	0·013
Albumin (g/dL)	3·5 ± 0·99	4·2 ± 0·98	0·049	3·6 ± 0·9	3·9 ± 1·1	0·041
WBCs (×10^9^/L)	4·7 ± 1·5	5·8 ± 1·5	0·111	4·6 ± 1·3	5·9 ± 1·3	0·004
Platelet count (×109/L)	164·8 ± 25·7	153·8 ± 27·7	0·647	149·7 ± 26·6	152·0 ± 30·9	0·908
**Treatment with sofosbuvir, ribavirin, and pegylated interferon**						
**Variables**	^*^IL-28B CC genotype		***P*** **value**	^*^IL-28B T allele carriers		***P*** **value**
	**Before**	**After**		**Before**	**After**	
**Without zinc administration**						
ALT (U/L)	41·0 ± 12·5	35·3 ± 9·5	0·461	44·3 ± 11·4	42·2 ± 11·1	0·530
AST (U/L)	43·3 ± 12·1	32·7 ± 8·95	0·032	44·9 ± 12·8	37·4 ± 11·3	0·008
Bilirubin (mg/dL)	1·5 ± 0·2	1·0 ± 0·2	0·033	1·0 ± 0·17	0·7 ± 0·6	0·884
Albumin (g/dL)	3·4 ± 0·8	3·9 ± 0·8	0·084	4·1 ± 1·1	4·3 ± 1·2	0·664
WBCs (×10^9^/L)	5·1 ± 1·4	4·5 ± 0·96	0·497	5·8 ± 1·3	3·74 ± 0·9	0·004
Platelet count (×109/L)	150·8 ± 31·2	166·0 ± 33·6	0·647	201·5 ± 33·3	164·1 ± 29·5	0·908
**With zinc administration**						
ALT (U/L)	49·8 ± 10·9	36·8 ± 9·4	0·011	47·2 ± 11·5	33·2 ± 9·6	0·024
AST (U/L)	50·4 ± 12·2	34·4 ± 11·4	0·017	63·5 ± 16·1	40·7 ± 11·6	0·009
Bilirubin (mg/dL)	1·4 ± 0·09	0·75 ± 0·03	0·036	1·30 ± 0·6	0·70 ± 0·2	0·337
Albumin (g/dL)	3·2 ± 0·9	4·2 ± 1·1	0·002	3·2 ± 0·82	4·3 ± 1·1	0·004
WBCs (×10^9^/L)	6·2 ± 1·1	4·9 ± 1·0	0·061	6·9 ± 1·5	4·5 ± 1·1	0·0008
Platelet count (×109/L)	200·3 ± 32·9	197·4 ± 25·4	0·890	213·5 ± 34·9	173·3 ± 24·4	0·030

Abbreviations: *AST* aspartate aminotransferase, *ALT* alanine aminotransferase, *WBCs* white blood cells. ^*^IL-28B *rs12979860* single-nucleotide polymorphism genotypes. *T allele carriers* CT/TT combined. *P* < 0·05 is considered significant. Continuous variables were expressed as mean ± SD

### IL28B C/T SNP and zinc administration affect fibrosis outcome after treatment

**Fig. 2:**
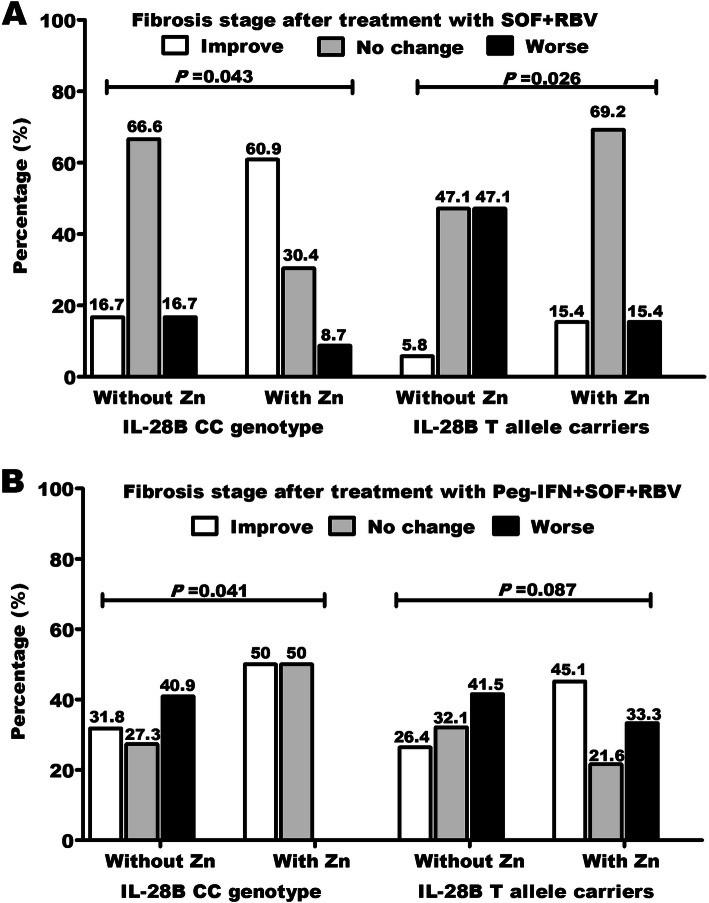
Zinc administration improve liver stiffness (decrease fibrosis grade). Zinc administration during treatment with both **A** SOF/RBV and **B** SOF/RBV + Peg-INF improve fibrosis in HCV patients. Fibrosis improvement was more noticeable in patients with CC genotype than T allele carriers. IL-28B rs12979860 single-nucleotide polymorphism genotypes. T allele carriers = CT/TT combined

**Fig. 3:**
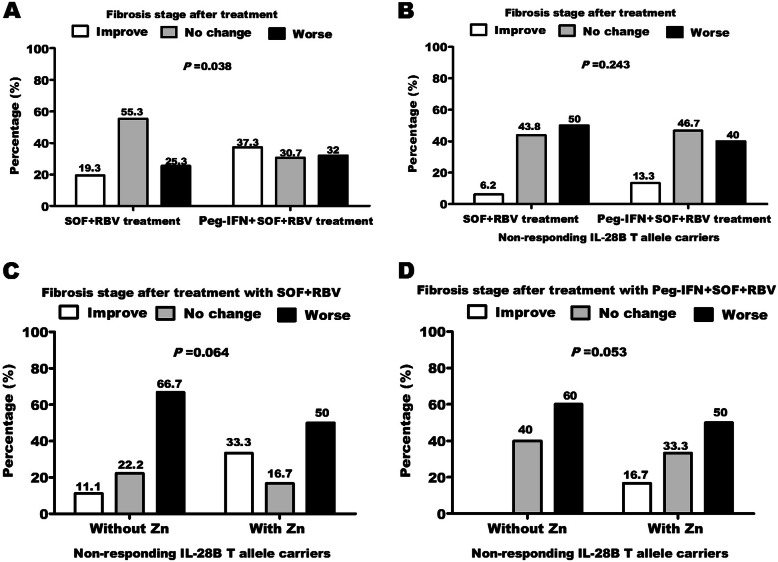
A comparison of dual vs. triple antiviral therapy. **A** Fibrosis improvement was more noticeable (*P* = 0.038) in patients treated with SOF/RBV than patients treated with SOF/RBV + Peg-INF. Regarding only non-responders of T allele carriers, **B** triple treatment (SOF/RBV + Peg-INF) decrease patient’s liver stiffness more efficiently than dual treatment and also zinc administration decrease patient’s liver stiffness in both **C** SOF/RBV and **D** SOF/RBV + Peg-INF treatments

Hepatic fibrosis was improved in 16.7% of patients with CC genotype in contrast to 5.8% of patients with CT/TT genotypes after treatment with SOF/RBV without zinc administration (Fig. 2A). In contrast, treatment with SOF/RBV + Peg-INF improved hepatic fibrosis in 31.8% and 26.4% in CC and CT/TT, respectively (Fig. 2B). Interestingly with zinc administration, improved fibrosis reached 60.9% and 50% in patients with CC genotype after treatment with dual and triple regimens, respectively. In contrast, hepatic fibrosis improved to 15.4% and 45.1% after treatment with dual and triple regimens, respectively in patients with CT/TT genotypes. The improvement in hepatic fibrosis was associated with IL28B CC genotype, zinc supplementation, pre-treatment early fibrosis stages (Table 2), and triple therapeutic regimen (Fig. 3A).

**Table 2:** The change in fibrosis grades before and after HCV treatments according to FibroScan

	**Fibrosis stage after treatment with SOF + RBV**													
	**Without zinc administration**													
	^*^ **IL-28B CC genotype**							^*^ **IL-28B T allele carriers**						
**Fibrosis stage before treatment**	**Stage**	**No**	**F0**	**F1**	**F2**	**F3**	**F4**	**Stage**	**No**	**F0**	**F1**	**F2**	**F3**	**F4**
	**F0**	4	3	1	0	0	0	F0	3	2	1	0	0	0
	**F1**	6	1	3	1	1	0	F1	2	0	1	1	0	0
	**F2**	8	0	2	5	1	0	F2	9	0	0	3	6	0
	**F3**	3	0	0	1	2	0	F3	23	0	2	0	6	15
	**F4**	3	0	0	0	0	3	F4	13	0	0	0	1	12
	**With zinc administration**													
	**Stage**	**No**	**F0**	**F1**	**F2**	**F3**	**F4**	**Stage**	**No**	**F0**	**F1**	**F2**	**F3**	**F4**
	**F0**	7	6	1	0	0	0	F0	5	4	1	0	0	0
	**F1**	7	6	0	1	0	0	F1	4	1	3	0	0	0
	**F2**	4	2	2	0	0	0	F2	6	0	1	4	1	0
	**F3**	2	0	1	1	0	0	F3	15	0	0	0	9	6
	**F4**	3	0	0	0	2	1	F4	19	0	0	0	6	13
	**Fibrosis stage after treatment with Peg-IFN + SOF + RBV**													
	**Without zinc administration**													
	^*^ **IL-28B CC genotype**							^*^ **IL-28B T allele carriers**						
	**Stage**	**No**	**F0**	**F1**	**F2**	**F3**	**F4**	**Stage**	**No**	**F0**	**F1**	**F2**	**F3**	**F4**
	**F0**	8	3	0	3	2	0	F0	10	1	8	1	0	0
	**F1**	7	4	0	3	0	0	F1	12	2	4	2	2	2
	**F2**	4	0	2	1	1	0	F2	7	0	2	4	1	0
	**F3**	1	0	0	0	1	0	F3	12	0	2	2	4	4
	**F4**	2	0	0	0	1	1	F4	9	0	0	2	3	4
	**With zinc administration**													
	**Stage**	**No**	**F0**	**F1**	**F2**	**F3**	**F4**	**Stage**	**No**	**F0**	**F1**	**F2**	**F3**	**F4**
	**F0**	4	4	0	0	0	0	F0	2	2	0	0	0	0
	**F1**	10	6	4	0	0	0	F1	15	6	2	4	3	0
	**F2**	4	2	2	0	0	0	F2	6	2	2	0	2	0
	**F3**	3	0	0	1	2	0	F3	17	0	6	3	0	8
	**F4**	3	0	0	0	1	2	F4	11	0	1	3	0	7

*IL-28B rs12979860 single-nucleotide polymorphism genotypes. *T allele carriers* CT/TT combined. Fibrosis grading was evaluated according to FibroScan: F0-F1: 2·5–6·9 kPa; F2: 7·0–9·4 kPa; F3: 9·5–12·4 kPa; F4: ≥ 12·5 kPa. *No* number of patients

### Zinc supplementation improved fibrosis in non-responding CT/TT genotypes

**Fig. 4:**
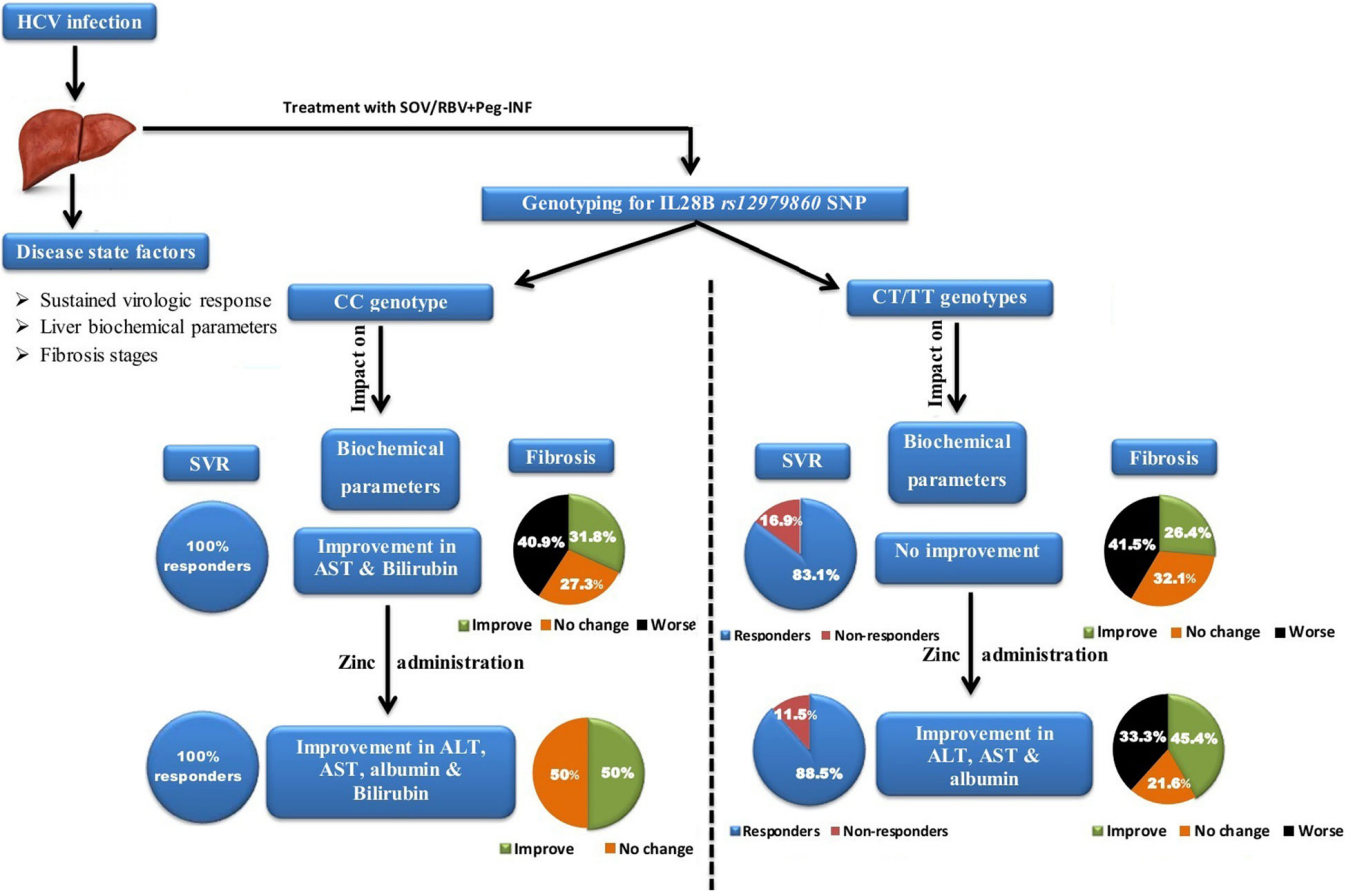
Study overview and key points. In this summarized figure, antiviral triple treatment (SOV/RBV + Peg-INF) was selected due to its favorable outcome on liver damage and fibrosis compared to dual treatment. Fibrosis state was evaluated by FibroScan. SVR: Sustained virologic response. Biochemical parameters are liver function tests including alanine and aspartate aminotransferase (ALT&AST), albumin, and total bilirubin

Improved fibrosis increased in triple more than dual regimens (Fig. 3B) in patients with CT/TT genotypes who did not respond to antiviral treatments (non-responders). In both dual (Fig. 3C) and triple (Fig. 3D) regimens, zinc supplementation had good effect on fibrosis outcome, the percentage of patients with improved fibrosis after treatment increased and worsened fibrosis decreased. Study overview and key points are summarized in Fig. 4.

## Discussion

The *rs12979860* genetic variant, located in IL28B gene, is a strong predictor of response to Peg-INF-based treatments. Compared to individuals with CT/TT genotypes, individuals with the favorable CC genotype have about a 2-fold higher likelihood of achieving SVR [14]. The American Association for the Study of Liver Disease recommends the use of SOF combination therapy as first-line therapy for the initial treatment of HCV-infected patients [15]. However, in specific treatment regimens that include SOF, the SVR was relatively high in all IL28B genotypes. The IL28B variant still influences treatment outcomes [4]. The 12-week SVR rate was 99% in individuals with CC genotype and 87% in individuals with non-CC genotypes [4]. Thus, the development and approval of more potent DAA therapy including INF-free regimens for hepatitis C has put into question the value and need of IL28B genotyping [16].

Exploring patient factors contributing to relapse between SVR and the end of treatment are needed [7]. So far, there are a few data regarding the role of IL28B *rs12979860* SNP as pharmacogenetic predictor during treatment with regimens that include SOF in Egyptian patients with HCV genotype 4 and it is still controversial. Here, in both SOF/RBV and SOF/RBV + Peg-INF therapeutic regimens, SVR was achieved in 100% of patients with CC genotypes in contrast to 84.5% and 85.6% in CT/TT carriers in SOF/RBV and SOF/RBV + Peg-INF regimens, respectively. In contrast to T allele carriers, we have found significant improvement in liver-related biochemical parameter in patients with CC genotype after antiviral treatments. On this basis, as predicted hepatic fibrosis was improved in higher percentage of patients with CC genotype compared with CT/TT genotypes after treatment with both dual and triple regimens.

IL28B variants also seem to influence treatment outcomes of regimens that include sofosbuvir in HCV genotypes other than 4. In patients with HCV genotype 1 infection and coinfection with HIV, the PHOTON trial reported that SVR rates were 80% in subjects with CC genotype and 75% in subjects with non-CC genotypes after treatment with SOF/RBV regimen [5]. Also, for the NETURINO study on patients with HCV genotype 1 or 4, most of them were with genotype 1. These patients were treated with SOF/RBV + Peg-INF regimen and it was reported that SVR rate was 99% in subjects with CC genotype and 87% in subjects with non-CC genotypes [4]. Also, in patients with IL28B non-CC genotype and infected with HCV-1a, Peg-INF-free regimens included combination of deleobuvir, faldaprevir, and ribavirin resulted in low SVR rates [17].

Hepatic function improvement, necro-inflammatory decrease, and fibrosis improvement were reported to be associated with SVR to DAAs [18]. Lower SVR rates to antiviral therapies are related to advanced fibrosis stages. Identification of predictive factors of fibrosis stages has implications for treatment optimization and selection [19]. Despite that many studies have yielded contradictory results on the association between fibrosis progression and IL28B genotype [13], it is not currently known how IL28B genotype could be a predictor of fibrosis improvement or aggravation after anti-HCV therapies and this is very important subject of debate. Interestingly in this study, improvement in hepatic functions and hepatic fibrosis was more frequent in patients with CC genotype compared to patients with CT/TT genotypes after treatment with both dual and triple regimens. Until now, the molecular mechanism controlling the association between IL28B SNPs and fibrosis progression in CHC patients has not been fully clarified [20]. Estrabaud et al. suggested that reduction of miR-122 expression is associated with IL28B unfavorable genotypes in patients chronically infected with HCV-1a [20].

In the second part of this study, we implicated zinc as flexible and powerful supplement to improve response to treatment regimens in T allele carriers and, more importantly, reduce or improve hepatic fibrosis and then increase survival and quality of life in CHC patients. In general, zinc supplementation improved hepatic functions and liver fibrosis in both CC genotype and T allele carriers. Also in T allele carriers, the rate of SVR was significantly higher in patients with zinc supplementation as compared to patients without zinc administration. Zinc deficiency may promote hepatic stellate cells activation, collagen production, and lipid peroxidation inhibition, causing increase in liver phospholipid content and liver fibrosis [21]. Also, zinc may inhibit hepatic fibrosis by reducing the activity of lysyl oxidase [22]. Our results may be explained also by the evidence provided by Read et al. [23] that zinc can act as specific potent inhibitor of IFN-λ3 signaling, that modulate chronic liver inflammation [24], and they highlight its potential as a therapeutic target for IFN-λ3-mediated chronic disease. Read et al. reported zinc inhibitory role on hepatic inflammation and suggested that hepatic zinc may modulate chemotaxis of dendritic cells, monocytes, and NK cells to inflamed tissues [23].

## Conclusions

The absolute SVR in patients with IL28B CC genotype and chronically infected with HCV genotype 4 support the selecting of these patients for shorter duration of treatment regimens and therefore this will be associated high economic value. This study highlighted the role of IL28B polymorphism in improvement of hepatic functions and fibrosis after antiviral treatments. Also, this study highlighted the introduction of zinc as a powerful supplement not only to increase SVR in non-responders but also to improve hepatic functions and fibrosis. Zinc supplementation is recommended particularly to patients with IL28B *rs12979860* T allele.

## Data Availability

Data not available due to [ethical/legal] restrictions: Due to research nature, the study participants did not agree for their data to be shared publicly, so supporting data is not available.
